# Mental health literacy among older adults in Shanghai: a descriptive qualitative study

**DOI:** 10.3389/fpsyg.2024.1470758

**Published:** 2025-02-07

**Authors:** Rongjing Xu, Mingrui Jing, Aining Zhang, Liqi Zha, Yan Wang, Anni Wang, Jun Tang, Biyun Xia, Shoumei Jia

**Affiliations:** ^1^Fudan University, School of Nursing, Shanghai, China; ^2^Huadong Hospital, Fudan University, Shanghai, China

**Keywords:** the aged, mental health, mental health literacy, qualitative research, thematic analysis

## Abstract

**Background:**

The aging population in China is surging rapidly, and elderly individuals are at higher risk of multiple mental health issues. Improving the mental health literacy of older adults can help them recognize mental illness and adopt proactive measures, potentially improving their mental health status and supporting the goal of healthy aging.

**Objective:**

To explore mental health literacy among older adults, providing a foundation for future interventions aimed at improving their mental health literacy.

**Methods:**

Guided by the new conceptualization framework of mental health literacy developed by Jiang et al. the study involved 20 community-dwelling older adults from four communities in Shanghai who were selected through purposive sampling to participate in semi-structured interviews. Thematic analysis was employed to summarize and extract themes from the data.

**Results:**

The qualitative analysis identified three primary themes and eight subthemes: inadequate knowledge about mental health and illnesses, negative intentions and attitudes toward maintaining mental health and preventing mental illnesses, and health behavior to promote mental health and prevent mental disorders.

**Conclusion:**

This study reveals significant gaps in mental health literacy among older adults, underscoring the necessity for multifaceted interventions. It calls for concerted efforts from individuals, families, and society to bolster mental health knowledge, challenge stigma, and encourage supportive behaviors. By integrating these approaches with the ‘Healthy China 2030’ policy, we aim to enhance mental health literacy for the aged.

## Introduction

The aging population in China is expanding rapidly. According to the seventh national population census data, individuals aged 60 and above have reached 246 million, representing 18.7% of the total population. Furthermore, those aged 65 and older number 191 million, accounting for 13.5% of the population (National Bureau of Statistics, 2021). Compared to younger individuals, older adults face increased health risks and are more susceptible to various mental health issues due to distinct physiological and psychological factors, as well as changes in family dynamics, and the erosion of traditional family structures, which are driven by population mobility (Zhang, 2020). Consequently, reduced physical capacity or disability among the aged often leads to mental health concerns, such as reduced self-esteem, which can result in withdrawal from social activities, further intensifying negative emotions (Huang et al., 2022). A recent study analyzing data from the Chinese Health and Longevity Database, which included 14,417 individuals aged 60 and above, reported an anxiety symptom detection rate of 12.15%. Additionally, a meta-analysis of 32 cross-sectional studies across China found that the combined prevalence of depressive symptoms among the elderly stands at 22.7%. Furthermore, a comprehensive national study covering 31 provinces revealed that the prevalence of dementia among those aged 65 and older in China is 5.6% (Fan et al., 2023). Globally, over 20% of individuals aged 60 and above experience mental health disorders, contributing to 6.6% of their total disease burden in terms of disability-adjusted life years (DALY) and 17.4% in years lost due to disability (YLD) (WHO, 2017). Despite this, the percentage of older adults seeking professional assistance for mental health concerns remains significantly low, largely due to a lack of mental health literacy within this demographic (Lu et al., 2019).

Mental health literacy (MHL) significantly affects mental health outcomes. Research has demonstrated that high mental health literacy can aid in the early recognition of mental illness, reduce stigma, and encourage suitable coping strategies, timely support, effective treatment, and improvements in mental health and well-being. Conversely, individuals with limited mental health literacy may rely on ineffective coping strategies, delay help-seeking, or abandon treatment prematurely due to stigmatizing attitudes (Kutcher et al., 2016b; Ren et al., 2020). Thus, improving mental health literacy represents one of the most basic, cost-effective, and impactful ways to improve individual mental health (Ming and Chen, 2020). The WHO asserts that mental health is just as crucial for older adults as it is at any other stage of life (WHO, 2017). While mental health literacy research has predominantly focused on children (Renwick et al., 2022), adolescents (Lu et al., 2023), married women (Lu et al., 2022), young adults (Jumbe et al., 2023), and healthcare workers (Wang et al., 2022), there has been comparatively limited attention to mental health literacy in older adults, highlighting the need for further research in this domain.

The concept of mental health literacy has been continuously evolving and improving over the past two decades through the contributions of various scholars and experts. In 1997, the concept of mental health literacy was first proposed by the Australian scholar Jorm et al. based on the concept of Health Literacy (HL). This concept refers to the knowledge and beliefs about mental illness that contribute to its recognition, management, and prevention (Jorm et al., 1997). Subsequent research on mental health literacy has largely been conducted based on the conceptual framework proposed by Jorm et al. In 2002, Sun et al. defined mental health literacy as ‘mental health awareness and ability, which encompasses the public’s knowledge, management, and prevention of psychosocial health issues (Sun et al., 2002); In 2009, Xiao et al. defined mental health literacy as “the individual’s ability to acquire and understand mental health knowledge and skills, to eliminate the stigmatization and discrimination against mental and psychological disorders, to identify, prevent, and manage mental and psychological diseases, thereby maintaining and promoting mental health (Li et al., 2009); In 2014, O’Connor et al. simplified the concept of mental health literacy into three dimensions: recognition, knowledge, and attitudes, with a particular focus on the knowledge aspect (O'Connor et al., 2014); In 2016, Kutcher et al. expanded the concept of mental health literacy to not only include stigma and knowledge of seeking help but also emphasized the importance of positive mental health literacy that focuses on the promotion of mental health (Kutcher et al., 2016a). It is apparent that the concept of mental health literacy proposed in the past has had its shortcomings to varying degrees. Since the concept of mental health literacy was initially introduced by psychiatrists, many of the definitions and theoretical research on mental health literacy, including those proposed by Jorm and others building on his work, have predominantly been framed within the context of psychiatry (Wu et al., 2021; Jorm, 2012). This focus has resulted in a concentration more akin to “mental illness literacy” rather than “mental health literacy,” with less emphasis on the broader aspects of mental health (Jorm et al., 1997; Wu et al., 2021). Additionally, nearly all studies emphasize individual mental health literacy, with scant attention given to the mental health literacy concerning others. Therefore, exploring a more comprehensive concept of mental health literacy is of significant importance.

**Figure 1:**
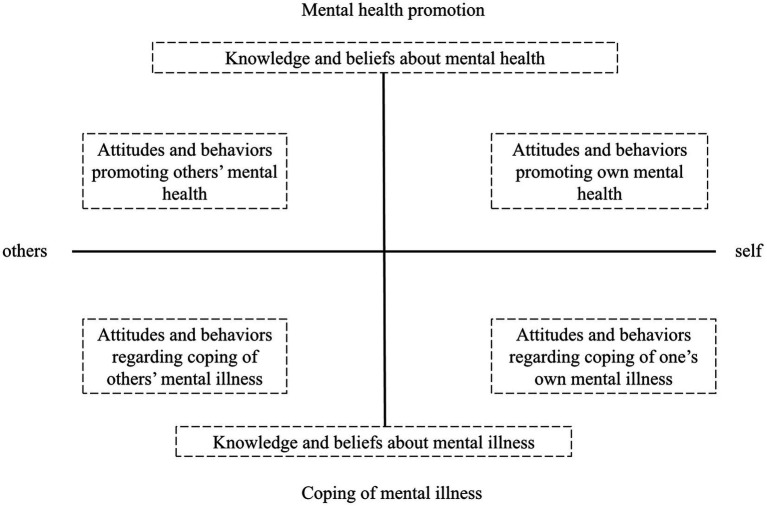
A new conceptual framework for mental health literacy. Reproduced with permission from Wu et al. (2023).

Jiang et al. (2020) introduced a novel, localized conceptual framework for indigenous mental health literacy, redefining it as “the knowledge, attitudes, and behaviors that individuals acquire to promote both their own mental health and that of others, as well as to manage their own and others’ mental illnesses.” This framework is organized into two dimensions: “mental health promotion and coping with mental illness” and “self and others.” It also includes three content aspects: knowledge, attitudes, and behaviors. The framework covers several facets: understanding mental health knowledge and concepts, understanding knowledge and concepts related to mental illness, engaging in behaviors that promote one’s own mental health, supporting the mental health of others, adopting behaviors and attitudes to address one’s own mental health issues, and supporting others with mental illness. These behaviors and attitudes can be viewed from the perspectives of “helping oneself” and “helping others” (Figure 1). Distinct from previous models, this conceptual framework goes beyond knowledge, attitudes, and behaviors associated only with mental illness, incorporating elements for mental health improvement. This broader perspective makes mental health literacy more comprehensive and cohesive. Recognizing the depth and inclusiveness of this concept, this study undertook a qualitative investigation to explore mental health literacy among older adults using this framework. The framework for mental health literacy (MHL) expands the traditional model by integrating knowledge, skills, and attitudes that apply to both self and others. While the framework offers a broad and holistic approach, there remains potential to refine its elements for greater specificity. This study aims to further enrich the new conceptual framework of mental health literacy.

Consequently, this qualitative study adopts the new conceptual framework proposed by Jiang et al. to explore the mental health literacy among older adults, focusing on three distinct dimensions: knowledge about mental health and psychological disorders, attitudes toward maintaining and promoting mental health and coping with mental illnesses, and behavioral habits related to the maintenance and enhancement of mental health and response to mental illnesses.

## Methods

### Study design

This study adopted a descriptive qualitative research approach, utilizing semi-structured, in-depth, one-on-one interviews with a sample of community-dwelling older adults. To enhance the rigor and transparency of the research process, the Consolidated Criteria for Reporting Qualitative Research (COREQ) checklist was adhered to throughout the study.

### Research setting and participants

Participants were selected through purposive sampling, adhering to the principle of maximum variation across factors such as age, gender, living arrangement, and marital status. Older adults from four Shanghai communities—Putuo District, Jing’an District, Hongkou District, and Minhang District—who were interviewed between July 2023 and March 2024 were included in the study. These districts, represent both urban and suburban settings, provided a diverse sample that enabled the exploration of mental health literacy differences among older adult residents across various geographical contexts in Shanghai. Hongkou District, for instance, has the highest proportion of residents aged 60 and above (44.9%), followed by Putuo District at 42.8%, Minhang District at 32%, and Jing’an Districts at 42.1%, respectively. These community settings were therefore selected to achieve a diverse sample. Inclusion criteria for participants included: (1) voluntary participation; (2) age of 60 years and older (≥60 years); and (3) residence in the community. Exclusion criteria included: (1) severe physical illness; (2) neurocognitive impairment or other psychiatric disorders; and (3) inability to communicate normally. The study protocol received approval from the Ethics Committee of a university’s School of Nursing (IRB#2023-4-2).

### Research team

The research team comprised two associate professors, two associate chief nurses, two graduate students, and three undergraduate students, all of whom were women. Team members received professional training in qualitative research prior to conducting interviews, with the first author undergoing one academic year of training. Further, the corresponding author has over 20 years of experience in geriatric psychological nursing education.

### Determine the outline of the interview

A preliminary interview outline was developed for this study, drawing from the new conceptual framework of mental health literacy proposed by Jiang Guangrong et al. This outline was developed by taking into account the characteristics of the aged and referencing interview outline from other similar studies (Khatib and Abo-Rass, 2022; Wu et al., 2023; Liu, 2018; Qiao, 2023). Following conducting preliminary interviews with two community elderly individuals, the outline was modified, and their interview results were excluded from the final analysis. After discussion within the research group, the final version was established (Table 1). To build rapport, the researcher used opening questions such as, “How have you been feeling lately?” and “What do you usually do to support your mental health?”

**Table 1:** Outline of qualitative research interviews.

Serial number	Outline of an interview questions
1	What ways do you think are conducive to older people’s mental health? Which ones are harmful?
2	What are, in your opinion, the indicators of good mental health and the symptoms of mental illness in older adults?
3	Can you tell us what psychological problems you know that older people are prone to? What do you know about Alzheimer’s disease or depression?
4	What are your views on promoting mental health in yourself and other older people?
5	How do you feel about older people with mental illness?
6	How do you maintain and promote your own and other older people’s mental health in your life?
7	What would you choose to do if you had a psychological problem of your own?
8	What would you do for elderly people around you who suffer from mental illnesses?

### Data collection

From July 2023 to March 2024, the researcher conducted semi-structured, face-to-face interviews with older adults in Shanghai. These interviews were held in private settings, chosen for participant comfort, often in community offices or participants’ homes, with privacy a priority. Before each session, participants provided informed consent and completed a general information questionnaire, fostering a trusting relationship. Audio recordings and field notes captured the interviews, with the researcher noting non-verbal cues to ensure the participants’ genuine thoughts were expressed without bias. Each interview, lasting 30 to 45 min, was organized and confirmed by participants post-session. The sample size was determined through thematic saturation, with data collection ceasing after 20 interviews when no new themes emerged, confirmed by two additional interviews.

### Data analysis

Data collation and analysis were conducted concurrently throughout the study. Interview durations ranged from 30 to 62 min, averaging 36 min, totaling 736 min. After processing, the dataset comprised 20 transcripts with approximately 88,024 words. Following each interview, the audio recordings were transcribed and promptly proofread by a professional within 24 ~ 72 h. Then, the transcripts were cross-checked and supplemented using field notes. For data analysis, two researchers independently applied thematic analysis (Braun and Clarke, 2014) through a manual coding process involving the following steps: (1) thorough and repeated reading of interview data for in-depth analysis; (2) identification and highlighting of significant words and phrases; (3) coding of keywords and phrases, followed by summarizing and categorizing codes with similar attributes; (4) initial identification of themes; and (5) reviewing, refining, defining, and naming themes. Occasionally, analysts encountered divergent interpretations of the text data. In such cases, extensive discussions focused on the nuances of data coding and theme formulation. If consensus remained unresolved, a third-party expert, typically a mentor, was consulted for guidance.

### Ethical considerations

The study was conducted in line with the *Declaration of Helsinki*, with design and methodology reviewed and approved by the ethics committee of the author’s university. All participants signed informed consent forms voluntarily prior to the study. The researcher explained the study’s purpose and importance and assured participants of confidentiality and privacy protection.

### Quality control measures

(1) The researchers, comprising graduate and undergraduate nursing students, received systematic training in qualitative research, equipping them with expertise in qualitative interview principles, skills, and procedures. Additionally, they conducted a literature review to familiarize themselves with relevant studies prior to the study’s commencement; (2) Interviews were conducted in a quiet, undisturbed environment; (3) During the interviews, researchers practiced “suspension,” refraining from imposing personal views or critically assessing participants’ statements; (4) Interview data were transcribed into text promptly (within 24 ~ 72 h) and verified with participants to ensure accuracy; (5) Two researchers independently analyzed the data to minimize subjective bias, with the final themes determined through group discussion.

## Results

The average age of participants was 73.05 ± 8.15 years. General demographic details of the respondents are provided in Table 2. Analysis of the interviews yielded three key themes: (1) inadequate knowledge about mental health and illnesses; (2) negative intentions and attitudes toward maintaining mental health and preventing mental illnesses, and (3) health behavior to promote mental health and prevent mental disorders.

**Table 2:** General information about the interviewees (*n* = 20).

Serial number	Age (years)	Gender	Residency Style	Educational level	Self-perceived physical condition	Self-perceived psychological condition	Existing health problems
E1	74	Female	①	Junior high school	Good	Good	Hypertension
E2	66	Female	①	Senior high school	Fair	Good	Bronchitis, diabetes, heart disease
E3	71	Female	②	Junior high school	Fair	Good	Diabetes, high blood pressure, heart disease, arthritis
E4	79	Female	③	Senior high school	Fair	Fair	Hypertension
E5	65	Female	①	College	Good	Fair	Bronchitis
E6	80	Female	③	College	Good	Good	Hypertension, arthritis
E7	67	Female	④	Technical secondary school	Fair	Good	Diabetes
E8	90	Male	⑤	Senior high school	Fair	Good	Bronchitis, lung cancer, bradycardia
E9	69	Female	①	Junior high school	Good	Good	None
E10	65	Female	①	Senior high school	good	good	Asthma
E11	70	Male	①	Junior high school	Fair	Good	Bronchitis
E12	68	Male	⑥	Senior high school	Good	Good	None
E13	80	Female	①	Primary school	Poor	Good	Hypertension, heart disease, primary bronchial lung cancer
E14	81	Male	①	College	Poor	Good	Bronchitis, osteoporosis
E15	80	Female	①	Technical secondary school	Poor	Good	Bronchitis, high blood pressure, heart disease, arthritis, osteoporosis
E16	82	Male	①	Primary school	Poor	Good	High blood pressure, arthritis
E17	78	Female	①	College	Fair	Good	High blood pressure, asthma, hip osteoarthritis
E18	61	Male	④	College	Fair	Good	Hyperlipidemia, emphysema, gout
E19	73	Male	①	College	Good	Good	Bronchitis, pneumonia, prostatitis
E20	60	Female	⑦	Primary school	Good	Good	None

①, Living with spouse; ②, Living with mother; ③, live alone; ④, Living with children and spouse; ⑤, Living with grandchildren; ⑥, Living with own children; ⑦, Other (living with the older person for whom they provide services).

### Inadequate knowledge about mental health and illnesses

#### Poor understanding of mental health and illnesses

We observed that some interviewees confused mental health with physical health, though older participants tended to believe there was a close relationship between the two (*E17: “In fact, to be honest, the body and mind are linked together; when the body is healthy, the mind is also healthy, and when the body is not healthy, the mind experiences many problems. When the ‘body’ is not healthy, the ‘mind’ is full of problems.” E18: “When the body is healthy, the mind feels healthy, but when the body is not healthy, there will be anxiety and worry.”*). However, when asked specifically about their mental health, participants often described it in terms of their own or others’ physical health conditions and related disease indicators, such as blood glucose and blood pressure levels (*E4: “I’m going to have an increase in all the indicators, blood glucose, blood pressure, etc.” E9: “Generally, I do not have diabetes or hypertension when my blood sugar and blood pressure are measured.”*).

In terms of characteristics displayed by mentally healthy older adults, respondents mentioned traits like looking younger than their age, being in good spirits, cheerful, mentally sharp, having a positive personality, willingness to confide in others, being relatively well-educated, self-sufficient, content, optimistic, confident in life, and capable of normal communication. *E1: “Some of them, although they look like they are in their eighties, have a good complexion and are cheerful, and generally, those like them are also relatively well-educated.” E2: “They all seem to be in a mental state where they feel cared for, living in less stressful conditions than in the past.” E3: “It depends on personal character; my mom has a good character, says what she has to say, does not hold things in, eats and sleeps well, and does not dwell on many issues.” E18: “He has a good mental outlook, walks with energy, appears optimistic; if he is 80 years old and still looks like he is 70, or even in his sixties, it proves he has confidence in life, a harmonious family, and filial children.”*

Regarding the characteristics exhibited by older adults suffering from mental illness, respondents described them as having many worries, frequently overthinking, experiencing memory problems, being stubborn, burdened by excessive thoughts, overusing their mental energy, feeling depressed, easily pessimistic, socially isolated, self-contained, bitter, avoiding social interaction, and having suicidal thoughts. *E2: “Worries, it’s just that it does not feel good here and there, and when you are older, you always have a pain somewhere.” E5: “He thinks a lot if he is really in a poor mental state.” E6: “For example, forgetfulness, he cannot remember things well, and sometimes forgets mid-conversation. I’ve heard that older people sometimes experience pain in a joint under their big toe, which is said to be a precursor to Alzheimer’s.” E8: “He is stubborn, thinks he’s right, tends to magnify small issues, and carries a heavy mental burden.” E9: “The more frequently someone engages in intense mental activity, the higher their risk of developing a related mental illnesses, often attributed to excessive brain use. In more severe cases, individuals with Alzheimer’s disease typically require assistance with eating; otherwise, they may be unable to eat properly.” E11: “When you look at an elderly person’s mental energy, you see it in the way they walk; a bent back often reflects poor spirit. You can also see it in their face. They may experience a very low mood, often tear up, and generally appear pessimistic.” E14: “They may show social withdrawal, avoid crowds, and refrain from communication.” E17: “Mentally unhealthy individuals experience a shrinking social circle and become increasingly detached from society.” E18: “Bitter and socially withdrawn, this type of person communicates minimally, avoids social activities, has few friends, and lacks someone to talk to, making them prone to anger and disrespect from others, potentially leading to depression.” E19: “They do not want to see anyone, prefer to stay behind closed doors, and may have thoughts of death.”*

#### Emphasis on describing mental illnesses through moral malpractice

Some interviewees associate mental health closely with moral character, perceiving mental ill-health as indicative of character flaws. When discussing mental health issues in those around them, many interviewees initially link it to moral conduct, frequently using morally charged terms such as “calculating,” “careful,” and “jealous” to describe individuals with mental illnesses, while overlooking other traits. *E3: “it is just small-mindedness and jealousy, because individuals with mental health issues perceive their family is not as well-off as the one next door.” E9: “Always wanting to get the upper hand, always trying to be above others…” E10: “An elderly female resident previously living in the downstairs apartment exhibited behaviors that were occasionally perceived as pretentious. So I believe she is suffering from psychological disorders.” E16: “Individuals with psychological disorders often have a tendency to judge others.” E18: “Mentally ill people are often snarky because they do not want to communicate properly… (frowning and waving hands).”*

#### Lack of systematicity in knowledge acquisition

In this study, older adults primarily gained knowledge of mental health and illness through online media (such as TV programs and cell phone notifications), books, and personal or observed experiences from those around them (including experiences and observations).

Some seniors indicated that witnessing mental health conditions in individuals within their community significantly deepened their understanding of mental health issues relevant to their age group. *E2: “There are* var*ious types of dementia, from those who are silent to those who are more active, and dementia often starts with not recognizing family members. For instance, my friend’s mother-in-law has dementia.” E10: “My father-in-law had dementia before. He would point to a spittoon and say he wanted a moon cake.” E11: “My former colleague, once active, became distant after experiencing mild depression and significant stress, transforming into a different person.” E13: “My husband, diagnosed with Alzheimer’s in July, is now bedridden and unconscious at home, only able to consume liquid food. Occasionally, he blinks and nods in response to our children calling him ‘Dad’ and asking for recognition.” E19: “My leader, managing his depression with imported medication, frequently voices a wish to die amid his intolerable suffering.”*

Some older adults acquire knowledge about mental health and illness through electronic sources, including cell phones and television. *E6: “I read it on the WeChat subscription account.” E8: “I regularly watch programs on preventing Alzheimer’s disease on TV show “Clinic X.”*

Others gain relevant information through books, lectures, and personal observations, sometimes speculating on the causes of illness based on patients they know. *E1: “Our community regularly organizes health lectures, occasionally featuring doctors from the local hospital who come to deliver talks. I make it a point to attend these classes whenever they are held.” E15: “Alzheimer’s disease is linked to atherosclerosis in seniors, causing thrombosis in blood vessels, which can lead to paralysis and neurological and linguistic impairments due to infarction or cerebral hemorrhage.”*

### Negative intentions and attitudes toward maintaining mental health and preventing mental illnesses

#### Weak willingness to reach out for support

Respondents showed a low willingness to reach out for support by discussing their feelings. Even if they were open to talking, it was mostly limited to immediate family members, often with a tendency to “report the good news but not the bad.” When older adults perceive themselves as experiencing mental health issues, they generally avoid seeking help from external sources. *E1: “Most psychological issues need to be dealt with internally; they are my personal matters, so there’s no need to share them with others.” E8: “If I had a mental illness, I would not seek help because I think my thoughts are unreasonable.” E10: “If I feel unhappy, instead of talking to others, I occasionally share my feelings with my children.” E16: “I talk to my wife and daughter about anything I encounter outside, but I generally avoid sharing things with people outside the family.” E18: “It’s difficult for an outsider to understand family matters. Many people treat it like a joke, so I keep it to myself.” E19: “I do not discuss my depression with my wife. Generally, people with depression, like myself, will not tell anyone, it’s a matter of privacy, and there’s a feeling of shame.”*

#### Feeling powerless

Some respondents expressed a desire to assist other elderly individuals, particularly those with mental illnesses. However, they reported feeling limited in their ability to provide such assistance due to various reasons. These include their own physical constraints, fear of harm, lack of familiarity with neighbors due to infrequent contact, and the reluctance of others to communicate, etc. *E4: “I cannot care for mentally ill elderly on my own, and my only concern is whether prices go up.” E13: “I live in a high-rise building and have difficulty with my legs and feet, so I am unable to care about the mental health of the elderly around me.” E16: “Because of my health, I only leave for social events and do not proactively support other seniors.” E18: Nowadays, residents in new housing complexes are unfamiliar with each other, have minimal interactions, and seldom even exchange basic greetings.*” *E18: “I try to be patient, and if I could see a way to help, I would assist older adults with mental health issues. But I worry that someone with Alzheimer’s might act unpredictably, throwing things, getting into fights, or even hurting me. This makes me have some concerns.” E19: “the doors of our the elderly neighbors are often closed, so even if we want to help, there’s no way we can.”*

#### Stigmatization tendency

A few older adults expressed some understanding of the negative impact of mental illness symptoms, acknowledging that “*sometimes he himself does not want this” (E20)*. However, the majority of respondents exhibited a tendency to stigmatize older adults with mental illness, often viewing them as personally responsible for their condition and expressing feelings of shame about mental illness. Many also indicated they would avoid individuals with mental health issues. *E4: “I stay away. And I cannot be friends with them.” E7: “Nowadays, many people suffer from depression, but if I were in their shoes, I would feel embarrassed to discuss it publicly and fear that others might laugh.” E8: “People with depression should recognize their own mistakes instead of blaming others; considering suicide as a response is a confrontational attitude, not a solution.” E12: “If an elderly person around me is always in a bad mood, I feel they might be in a poor state, so I keep my distance from them.”*

#### Health behavior to promote mental health and prevent mental disorders

Older adults demonstrate a range of health behaviors aimed at maintaining and promoting their own mental health. However, there is limited effort directed toward supporting and promoting the mental health of other elderly individuals, with a lack of depth in approaches for various reasons.

#### The diversity of ways to help oneself

Most interviewees typically use self-adjustment techniques to alleviate negative moods, such as changing their thoughts, redirecting their attention, etc. *E1: “I think that in order for someone to live a long life, they need to maintain a cheerful mood. In my usual experience, when moments of unhappiness arise, if I do not dwell on them, those feelings will pass by quickly.” E8: “I’m 90 years old and have lung cancer, but I accept it because we all have to adapt to the changes in our bodies.” E10: “When I become angry due to someone else ‘s actions, I try to put myself in their shoes and empathize with them. This method helps me and others to see the issue from different perspectives and allows me to calm down. E13: “I watch short videos on my phone every day; they distracts me from my negative moods.”*

Some respondents also choose to discuss their negative emotions with family members, believing that sharing with loved ones can relieve emotional burdens, effectively address issues, and prevent misunderstandings. *E10: “I might communicate with my children, it’s easier to resolve the matter.” E19: “I talked to my wife about my prostatitis, and it made me feel better.”*

Respondents enhance their mental health by cultivating diverse hobbies and engaging in various activities to bring enjoyment into their lives. *E1: “I used to sing, and I recently participated in a community event where I did a lot of clay sculpting, drawing, and other handicrafts” (displaying past handicrafts). E4: “I join the choir in the park, where I’ve made many good friends, and here are some photos from our events…” (showing the choir photos). E10: “The neighborhood committee offers a wonderful space equipped with air conditioning for hot days, where we can sing, laugh, chat, and do crafts—all activities that are beneficial for the brain.” E14: “Sometimes I play musical instruments, like the accordion and erhu, to shift my focus and lift my mood.” E20: “Every day, I exercise my fingers, stand on my tiptoes, and sunbathe. Just like cats, humans also need sunlight and stretching.”*

Some respondents also believe that socializing, building strong network connections, and maintaining good relationships can bolster mental health. *E7: “Engaging in conversation with others is beneficial; it’s important to interact and avoid being isolated.” E18: “Since retiring, I often chat and dine with some loquacious friends, sharing my inner thoughts.”*

#### Insufficient depth of helping other older persons

Some respondents expressed a general willingness to care for and check in on their elderly neighbors within the same building or community, helping to promote their mental well-being. *E3: “We all know each other here, upstairs and downstairs, and look out for each other.” E11: “We neighbors greet each other and check in on how each other has been doing lately.”*

Older adults also often offering support and encouragement to their peers can help them get rid of “bad moods.” *E1: “When neighbors quarrel, I step in and offer some advice.” E6: “I had a colleague worried about his son and daughter-in-law not having children after marriage; I advised him to stop obsessing over it, pointing out that they are now adults with his own life, and we should focus on our own lives instead.” E10: “When the weather’s nice, it’s lovely to have a group of us, maybe a dozen people, to sit outside in the sun and chat.” E14: “For those who get stuck in negative thinking, we try to encourage them to step out of it.”*

Many older adults also have reported their readiness to assist neighbors facing mental health challenges through small acts of kindness, demonstrating their care. *E1: “If they are facing financial difficulties, I am willing to help.” E9: “I could visit him, inquire about his needs, and offer assistance based on his response.” E18: “I assist with tasks that are challenging for him, such as carrying items or retrieving objects that have fallen from the balcony.” E11: “For example, if he is unable to lift heavy objects, I can assist him in transporting them home. I believe this provides assistance.”*

## Discussion

We utilized Jiang et al. ‘s new conceptual framework to explore the mental health literacy among older adults, which provided a constructive basis for our study. Our findings indicate that there is a pressing need to improve mental health literacy among the elderly.

### Knowledge gaps in the aged mental health literacy: bridging the divide

In this study, we primarily focused on two common mental illnesses in older adults, senile dementia and depression, to examine their knowledge in these areas. It founded that older adults possess a certain level of knowledge and personal experience regarding mental health and mental illnesses. For instance, they understand the interplay between mental and physical health and can provide vivid descriptions of the characteristics of individuals with mental health and psychological disorders, which is consistent with the results of two previous studies (Liu, 2018). But they may still harbor significant gaps in their understanding of mental health and mental illnesses. Older adults generally possess limited knowledge regarding the causes, prevention, and treatment of mental illnesses, echoing findings from previous studies (Lu et al., 2018; Zhou et al., 2021). Despite their ability to distinguish between mental health and mental illness, older adults struggled to identify specific symptoms that differentiate various mental illnesses, aligning with previous research findings (Piper et al., 2018).

This observed discrepancy in older adults’ knowledge and understanding of mental health and mental illnesses may arise from a multitude of factors. Firstly, age emerged as a significant factor; individuals under 70 demonstrated a higher level of mental health knowledge compared to those over 70. This result is consistent with prior research (Ding et al., 2022). Older adults with a college degree or above have a marginally better grasp of mental health and psychological disorders than those with different educational backgrounds. Secondly, cultural stigmas surrounding mental health issues can lead to a reluctance to discuss or seek help, perpetuating misunderstandings and a lack of awareness. Additionally, the absence of proactive efforts to acquire knowledge, possibly due to a lack of accessible resources or low health literacy, contributes to this gap. Community outreach and educational programs are often insufficient, failing to reach older adults effectively with vital mental health information. Furthermore, the media’s role in public health communication is underutilized, missing opportunities to educate a wider audience about mental health. These factors collectively hinder the dissemination of accurate mental health knowledge, leading to the persistent gaps in understanding observed in our study’s participants. Last but not least, it is evident that older adults may not perceive mental health with the same significance as physical health, which could be attributed to traditional beliefs and the prioritization of physical well-being over mental well-being. This indicates that, compared to physical health, mental health may not be accorded equal importance by this demographic.

In response to these findings and in alignment with the ‘Healthy China 2030’ policy and the ‘Healthy China Action Plan (2019–2030)’, which emphasize the importance of disease prevention, health promotion, and the enhancement of health literacy, future efforts should focus on enhancing community outreach and promotion of knowledge regarding mental health and psychological disorders. A specialized mental health curriculum could be developed to address knowledge gaps, incorporating peer mentorship programs that engage ‘experienced’ older adults as mentors, providing support and answering questions to facilitate peer-to-peer education. This approach not only responds to the need for increased health literacy but also aligns with the policy’s call for a high-quality, efficient, and integrated medical and health service system that promotes the improvement of health outcomes and reduces the burden of mental illnesses among older adults, contributing to a healthier and more informed society.

### Fostering positive attitudes toward mental health and mental illnesses in the aged

This study found that older adults typically hesitate to disclose their mental health concerns and psychological disorders to others, showing a reduced propensity to seek support in their daily lives. Even when they seek support, family is usually their first choice. This is similar to previous research findings (Xu et al., 2022; Xu et al., 2014). This phenomenon is likely driven by multiple underlying reasons: Firstly, many older adults are accustomed to dealing with problems on their own and are often disinclined to seek help, as they perceive it as an imposition on others. This reluctance to trouble others is likely rooted in the cultural context of their upbringing, where self-reliance was a strongly promoted value. Secondly, older adults may not recognize the significance of mental health issues or be unsure of when to seek professional assistance. They might regard psychological problems as less severe than physical illnesses and thus not feel the urgency to seek support promptly. Additionally, in many cultures, including those in Asia, the family is the predominant source of support and care (Kim et al., 2018; Campos et al., 2014; Xu et al., 2024). Consequently, older adults are more inclined to seek help from their family members, who constitute their most intimate and reliable social network. Finally, older adults may exhibit skepticism toward professional mental health services, citing concerns about the quality of care and privacy, which leads to a lower acceptance of such specialized services.

Despite no significant differences in attitudes toward mental health and psychological disorders among older adults with varying characteristics such as age, gender, marital status, and living arrangements, there are key points that merit attention: even those with higher levels of education may still exhibit refusal to seek help when faced with psychological illnesses and stigmatize such conditions. This suggests that the negative attitudes of older adults toward mental health and psychological disorders may be more deeply rooted in societal and cultural perceptions rather than being solely a matter of individual education levels. In Asian cultures, including our own, there exist negative stereotypes about psychological disorders, such as viewing those with mental illnesses as morally flawed or having made mistakes (Chang, 2007). Stigmatization can lead individuals with psychological disorders to conceal their mental health issues to avoid discrimination, refraining from seeking help or delaying medical attention (Fu and Ning, 2020; Xu et al., 2022).

Examining the attitudes of older adults toward the maintenance and enhancement of others’ mental health and the management of potential or existing psychological disorders, it is observed that those with chronic conditions exhibit a weaker desire to assist their peers compared to healthier seniors. Several reasons may account for this: Firstly, chronic diseases may cause physical fatigue or pain, limiting their ability to participate in social activities and provide assistance. Secondly, they might be more preoccupied with their own health issues, making it challenging to allocate energy and attention to others. Lastly, the need for increased rest and medical attention could lead to a reduction in their capacity to care for and support others.

To enhance support for older adults, future initiatives could establish community-based groups and centers. Training for family members in mental health support will empower them to better understand and assist elderly relatives. Improving the quality and accessibility of mental health services is vital, ensuring that older adults can easily access care with confidence. For those with negative perceptions of psychological disorders, altering their views to see these conditions as treatable, like common illnesses, is a prerequisite for effective intervention and building trust in mental health care. Special approaches can be adopted for older adults with limited mobility. In the digital era, short videos serve as a means for older adults to remain informed and connected, facilitating mental health improvements as suggested by research (Liu and Wang, 2024). Subsequent studies may leverage these videos to clarify misconceptions and promote awareness about mental health, emphasizing the significance of professional psychological support.

### Motivating and enhancing behavioral habits for older adults in mental health preservation and mental illness management

From the perspective of behaviors that older adults “help themselves” in maintaining and promoting mental health and coping with existing or potential psychological disorders, most elderly individuals, irrespective of gender and educational background, practice diverse behaviors conducive to mental health promotion, even without a full understanding of the underlying mechanisms.

There may be several reasons for this: Firstly, when participating in activities that promote mental health (such as stretching ligaments, taking walks, self-relaxation, and crafting), older adults may directly experience positive physical and emotional changes, such as relaxation, stress reduction, and increased vitality. Secondly, many older adults may have been practicing these activities since childhood. The power of habit ensures that they continue to engage in these activities even without a full understanding of the scientific basis of mental health. Lastly, community centers, senior activity groups, and family members may encourage older adults to participate in these activities, highlighting their benefits for mental health. This reinforcement motivates them to continue these practices. Additionally, the aged, particularly those with advanced age and multiple chronic conditions, often modify their previously established behaviors for mental health promotion to simpler alternatives, such as reading, finger exercises, and watching television and mobile videos, and so on. This may be related to the decline in their physical functions and the negative emotions associated with long-term chronic diseases. These factors could lead to a loss of confidence in engaging in complex behaviors that promote mental health; Self-rated individuals with good mental health tend to maintain and promote their mental well-being through diverse means compared to those who rate their mental health as average or poor. This may be attributed to the fact that older adults with a positive self-assessment of mental health often possess a more optimistic outlook on life, along with a stronger sense of self-efficacy and cognitive function, enabling them to engage in more effective self-regulation and stress management (Peng et al., 2024; Zhu et al., 2023).

Therefore, future initiatives can inspire older adults with self-rated average or poor mental health to adopt behaviors that promote mental well-being through community activities, health education, and positive feedback. For those who have already demonstrated a diversity of mental health promotion behaviors, it is important to explore how to help them understand and recognize the positive impact of these behaviors on their mental health, thereby motivating them to continue these habits. Considering that the elderly with advanced age and multiple chronic conditions may require simpler and more manageable ways to maintain their mental health, future research can investigate which simplified behavioral habits are most suitable for this group and assess the effectiveness of these habits.

From the perspective of behaviors where older adults “help others” in maintaining and promoting mental health and coping with existing or potential psychological disorders, it is observed that elderly individuals can assist their peers in everyday small acts, yet these actions lack depth. Elderly individuals show care and support by assisting with carrying personal items, which alleviates practical issues and may positively affect mental health. However, such help is limited by personal capabilities and may not address deeper psychological needs. Group activities like outdoor chatting enhance community cohesion and mental well-being but are subject to weather, health, and personal preferences, and may not be accessible to all seniors in need.

Therefore, specialized mental health services are indispensable. Communities can organize mental health lectures and consultation activities to provide older adults with opportunities for psychological counseling and communication, thereby supplementing their knowledge of the effects of healthy behaviors on mental health and enhancing their motivation to adopt and maintain such behaviors. Additionally, the community-based mutual aid model of elderly care, as an innovative approach, combines the advantages of family and social elderly care, facilitating the realization of the value of the elderly community. This model can promote collective participation and mutual service among the elderly, enriching their social experiences and spiritual lives (Zhou et al., 2023; Yu et al., 2024). Moreover, peer support may enhance the mental health of older adults and improve their anxiety and depression (Shalaby and Agyapong, 2020; Tang et al., 2022). Consequently, communities can organize more activities for the elderly, providing platforms and opportunities for them to offer psychological support to each other, thereby helping to further stimulate behaviors among older adults that promote the mental health of others. Looking ahead, community mental health service organizations can build long-term cooperative relationships with schools, hospitals, and other social groups on the basis of basic mental health services, to provide sustainable psychological support for the elderly. This can help address the lack of depth in behaviors through which older adults promote the mental health of others and cope with psychological diseases.

## Conclusion

This study employs the new conceptual framework proposed by Jiang et al. to explore mental health literacy among older adults, contributing new insights to the field and reinforcing the theoretical underpinnings of this important area. Our findings reveal significant gaps in mental health literacy, which we have identified across three key areas: Firstly, It has revealed a clear imperative to bolster mental health knowledge. Secondly, the research has underscored the pervasive influence of cultural stigmas on help-seeking behaviors and the tendency of older adults to rely on family for support, reflecting deep-seated societal attitudes toward mental health. Thirdly, while older adults engage in a variety of behaviors to promote their own mental health, their actions to support others’ mental well-being are often simpler and less comprehensive, suggesting a need to encourage and educate them on the benefits of these practices. In conclusion, addressing the knowledge gaps, challenging negative attitudes, and fostering proactive behavioral habits are essential for enhancing the mental health of older adults. Future initiatives should focus on community-based education, peer mentorship, and family involvement to create a supportive environment that aligns with the ‘Healthy China 2030′ policy. By doing so, we can empower older adults to better manage their mental health, reduce the stigma associated with mental illnesses, and promote a more integrated approach to mental health care that considers the unique needs and perspectives of this demographic.

### Strengths and limitations

This study stands out among mental health literacy research on older adults as one of the few qualitative investigations, involving both healthy and ill community-dwelling older adults in Shanghai. The study’s diverse sample and use of a conceptual framework aligned with the national context provided a solid foundation, effectively guiding the research to meaningful insights.

A limitation of this study is that it only focused on the mental health literacy of older adults in Shanghai, which may limit the applicability of the findings to other cities or populations. Future studies and validation efforts across other cities and nationwide are necessary to broaden the findings. Additionally, there may be differences in how older adults perceive methods for improving mental health. For instance, some participants may engage in mental health-promoting activities (e.g., attending community lectures, square dancing) without realizing their benefits for mental health, potentially leading to an underestimation or omission of these activities in the findings. Lastly, the study predominantly included talkative participants who may naturally have a positive outlook on life. This tendency could suggest higher levels of mental health literacy in behavioral habits, possibly influencing the study’s outcomes.

## Data Availability

The raw data supporting the conclusions of this article will be made available by the authors, without undue reservation.
